# 2452: Artificial urinary sphincter failure: Characterizing the causes of failure and individual device component survival

**DOI:** 10.1017/cts.2017.279

**Published:** 2018-05-10

**Authors:** Arnav Srivastava, Gregory Joice, Madeline Manka, Nikolai Sopko, Edward Jamie Wright

**Affiliations:** Johns Hopkins University School of Medicine, Baltimore, MD, USA

## Abstract

OBJECTIVES/SPECIFIC AIMS: Stress urinary incontinence (SUI) significantly affects quality of life and occurs in 60% of men after radical prostatectomy, with 5% requiring surgical treatment. The artificial urinary sphincter (AUS) offers these patients excellent control of their post-prostatectomy SUI. The device contains 3 parts: the pump, urethral cuff, and pressure regulating balloon. Despite the effectiveness of AUS, up to 50% of patients require surgical revision after initial placement due to recurring SUI. Thus far, literature is heterogeneous regarding the causes of mechanical AUS failure and appropriate surgical management. Our study aims to characterize the most common reasons of AUS failure requiring surgical revision and the survival of each AUS component. METHODS/STUDY POPULATION: We report a series of 48 patients who received AUS placement and/or revision by 1 surgeon from 2010 to 2013. Upon presenting for revision, intraoperatively, the surgeon systematically evaluated the device for failure of the balloon, cuff and pump as well as urethral erosion and atrophy. In patients not requiring revision all device components were presumed functional. We conducted retrospective chart review to collect baseline characteristics, intraoperative findings, and postoperative outcomes. Using Kaplan-Meier estimates, we calculated incidence rates of component failure for the cuff, pump, and balloon. To identify risk factors for AUS failure, Cox regression was performed for univariate and multivariable testing. Multivariable modeling included those variables considered biologically plausible and significant in univariate testing. RESULTS/ANTICIPATED RESULTS: In total, 48 patients were studied with median follow up of 4.25 years. All patients received an AMS 800 device with a 61–70 mL balloon filled with 27 cc of isotonic contrast. Cuff sizes ranged from 3.5 to 5.5 cm, with 4.5 cm selected in 33/48 cases (68.8%); 19 of the patients required AUS correction (41.7%). Balloon leak constituted 57.9% (11/19) of failures, followed by cuff failure/urethral atrophy (21.1%), urethral erosion (10.5%), and individual cases of infection and pump failure. Median time to mechanical failure due to balloon leak was 3.67 years (IQR 2.17, 5.33); median time to failure for nonballoon causes was 0.54 years (IQR 0.25, 1.83). Survival of the balloon, cuff, and pump was 100%, 95.7%, and 97.9% at 1 year and 76.9%, 91.0%, and 97.9% at 5 years, respectively. DISCUSSION/SIGNIFICANCE OF IMPACT: Our study identifies fluid leakage from the balloon as the most common cause of AUS failure, particularly in patients presenting between 1 and 5 years after initial placement. For such patients, interrogating the balloon first can decrease infection risk and surgical morbidity as it can avoid manipulation of the urethral cuff. Furthermore, simply replacing lost fluid saves cost and allows for immediate reactivation of the AUS device.

